# Ice age fish in a warming world: minimal variation in thermal acclimation capacity among lake trout (*Salvelinus namaycush*) populations

**DOI:** 10.1093/conphys/cou025

**Published:** 2014-07-16

**Authors:** Nicholas I. Kelly, Gary Burness, Jenni L. McDermid, Chris C. Wilson

**Affiliations:** 1 Environmental and Life Sciences Graduate Program, Trent University, Peterborough, ON, Canada K9J 5G7; 2 Department of Biology, Trent University, 2140 East Bank Drive, Peterborough, ON, Canada K9J 7B8; 3 Wildlife Conservation Society Canada, Trent University, Peterborough, ON, Canada K9J 7B8; 4 Ontario Ministry of Natural Resources, Trent University, Peterborough, ON, Canada K9J 8M5

**Keywords:** Climate change, metabolic rate, physiology, *Salvelinus namaycush*, temperature, thermal acclimation

## Abstract

In the face of climate change, the persistence of cold-adapted species will depend on their adaptive capacity for physiological traits within and among populations. The lake trout (*Salvelinus namaycush*) is a cold-adapted salmonid and a relict from the last ice age that is well suited as a model species for studying the predicted effects of climate change on coldwater fishes. We investigated the thermal acclimation capacity of upper temperature resistance and metabolism of lake trout from four populations across four acclimation temperatures. Individuals were reared from egg fertilization onward in a common environment and, at 2 years of age, were acclimated to 8, 11, 15 or 19°C. Although one population had a slightly higher maximal metabolic rate (MMR), higher metabolic scope for activity and faster metabolic recovery across all temperatures, there was no interpopulation variation for critical thermal maximum (CTM) or routine metabolic rate (RMR) or for the thermal acclimation capacity of CTM, RMR, MMR or metabolic scope. Across the four acclimation temperatures, there was a 3°C maximal increase in CTM and 3-fold increase in RMR for all populations. Above 15°C, a decline in MMR and increase in RMR resulted in sharply reduced metabolic scope for all populations acclimated at 19°C. Together, these data suggest there is limited variation among lake trout populations in thermal physiology or capacity for thermal acclimatization, and that climate change may impact lake trout populations in a similar manner across a wide geographical range. Understanding the effect of elevated temperatures on the thermal physiology of this economically and ecologically important cold-adapted species will help inform management and conservation strategies for the long-term sustainability of lake trout populations.

## Abstract (precis)

Cold-adapted fishes are increasingly vulnerable to warming conditions under climate change. We investigated how elevated temperatures impact the metabolic and thermal performance of lake trout populations. All populations showed similarly limited acclimation responses, suggesting that global warming will significantly threaten lake trout at the species rather than population level.

## Introduction

Global climate change is predicted to impact ecosystems significantly over the next century (Magnuson *et al.*, 1997; Schindler, 1997; Pörtner, 2002; Brander, 2010), with expected implications for species and populations (Walther *et al.*, 2002; Parmesan, 2006; Eliason *et al.*, 2011; Pauls *et al.*, 2013). Climate models project a global increase in average atmospheric temperature by 3.5–4.2°C over the next 50 years (IPCC, 2007). A change in both the average temperature and the thermal heterogeneity of terrestrial and aquatic environments will expose many populations to suboptimal conditions. Predicted effects include changes in the geographical distribution of species (Perry *et al.*, 2005; Pinsky *et al.*, 2013), alterations to phenological processes (Bradshaw and Holzapfel, 2006; Shuter *et al.*, 2012) and species interactions (Tylianakis *et al.*, 2008). Overall, this may potentially result in the extinction or extirpation of many terrestrial, marine and aquatic species over the next century (Thomas *et al.*, 2004; Xenopoulos *et al.*, 2005; Somero, 2010).

Freshwater ecosystems are considered to be particularly vulnerable to climate change (Magnuson *et al.*, 1997; Schindler, 1997; Ficke *et al.*, 2007; McCullough *et al.*, 2009). A chronic increase in atmospheric temperature is predicted to impact the thermal properties of freshwater lakes and their resident biota, with elevated epilimnetic temperatures and increased magnitude and duration of thermal stratification reducing the availability of suitable thermal habitats for cold-adapted species (De Stasio *et al.*, 1996; Stefan *et al.*, 1998; Ficke *et al.*, 2007). Many cold-adapted populations will be exposed to temperatures above their thermal optimum, which will increase energetic demands and drive selection on physiological traits to maximize performance at the new environmental temperatures (Stockwell *et al.*, 2003; Somero, 2010; Hoffmann and Sgro, 2011). Consequently, coldwater species may become extirpated from much of their present range (Casselman, 2002; Chu *et al.*, 2005). The persistence of cold-adapted species and populations may therefore be determined by their capacity to cope with or adapt to elevated temperatures, which may be constrained by limited genetic resources originating from finite ancestral populations in glacial refugia and post-colonization restrictions on local population sizes (Bernatchez and Wilson, 1998; Willi *et al.*, 2006).

In a changing environment, the ability to maintain performance over a range of environmental conditions determines the persistence of populations and species (Stillman, 2003; Hoffmann and Sgro, 2011). Phenotypic plasticity (e.g. acclimatization) allows individuals to adjust physiological performance over a range of environmental conditions, which can enhance fitness in an unstable environment (Wilson and Franklin, 2002; Somero, 2010). For many species, the capacity of local populations to buffer the negative effects of temperature change through thermal acclimatization will determine their persistence over the longer time periods required for evolutionary adaptation to changing climatic conditions (Stillman, 2003; Calosi *et al.*, 2008; Somero, 2010, 2011; Seebacher *et al.*, 2012). Relatively few studies have assessed variation in thermal acclimatization capacity among intraspecific populations, but the limited evidence suggests that thermal acclimatization for physiological traits may vary among conspecific populations (Lucassen *et al.*, 2006; Sylvestre *et al.*, 2007; Seebacher *et al.*, 2012). Understanding the degree of variation within and among populations for physiological traits, as well as the capacity of these traits for thermal acclimatization, is an important knowledge gap for identifying the potential impacts of climate change on cold-adapted species.

Previous investigations of the degree of interpopulation variation in the thermal physiology and acclimatization capacity of salmonid species have yielded conflicting results. Intraspecific variation in thermal physiology has been reported among populations of sockeye salmon (*O. nerka*; Lee *et al.*, 2003; Eliason *et al.*, 2011), cutthroat trout (*Oncorhynchus clarkia pleuriticus*; Underwood *et al.*, 2012) and brook trout (*Salvelinus fontinalis*; McDermid *et al.*, 2012). In contrast, multiple studies suggest that the thermal performance of salmonids remains highly conserved among populations (Jensen *et al.*, 2000; Rodnick *et al.*, 2004; Larsson *et al.*, 2005; Elliott and Elliott, 2010). However, most studies have not investigated interpopulation variation in thermal physiology and acclimatization capacity while using a common rearing environment to control for environmental and/or maternal environmental effects.

The aim of this study was to determine the thermal acclimation capacity of different populations of lake trout (*Salvelinus namaycush*), reared in common environmental conditions from egg fertilization onwards. In this way, we could minimize the influence of early environmental effects on thermal acclimation capacity (e.g. Scott and Johnston, 2012). The lake trout is a cold-adapted, stenothermal salmonid that evolved in response to the Pleistocene glaciations (Martin and Olver, 1980; Wilson and Hebert, 1996, 1998) and provides an excellent model species to investigate the acclimatization capacity of cold-water fish species. The optimal temperature for lake trout growth is 8–12°C (Christie and Regier, 1988), and their aerobic metabolic scope peaks at ∼15°C (Gibson and Fry, 1954; Evans, 2007). There is evidence of interpopulation variation in temperature preference for lake trout (McDermid *et al.*, 2013); however, the extent of variation in physiological traits in this species is largely unknown. During summer thermal stratification, lake trout take refuge from high epilimnetic temperatures in the colder hypolimnion of temperate and subarctic lakes (Mackenzie-Grieve and Post, 2006; Plumb and Blanchfield, 2009). Climate change is predicted to impact populations of lake trout and other coldwater species through a loss of optimal thermal habitat and exposure to suboptimal temperatures (Ficke *et al.*, 2007).

We tested the hypothesis that the capacity for thermal acclimatization differs among populations of a cold-adapted fish species. Individuals from four allopatric lake trout populations were reared in a common environment from egg stage. At ∼2 years of age, fish from each population were acclimated to four temperatures (8, 11, 15 and 19°C). We compared upper thermal resistance, routine (RMR) and maximal metabolic rates (MMR), metabolic scope for activity (MMR minus RMR), the time until exhaustion during a chase protocol and metabolic recovery from exhaustive exercise among populations, as well as describing their phenotypic thermal acclimation capacity and response to an extreme high-temperature challenge. In addition, we tested for acclimation temperature and population effects on body size (mass and fork length) and condition.

## Materials and methods

These studies were conducted in accordance with the guidelines of the Canadian Council on Animal Care and were approved by the Institutional Animal Care Committee of Trent University (Protocol # 22261) and the OMNR Aquatic Animal Care Committee (Protocol # 92).

### Lake trout populations

This study used four lake trout populations that were founded from wild inland populations across Ontario, Canada. Although it would have been desirable to assess variation among populations from across the species range with both geographical and phylogenetic representation, this was not possible due to logistical limitations on transporting fish or gametes across jurisdictional (provincial/state/federal) borders.

Two populations (Lake Louisa and Opeongo Lake) were established one generation ago from wild spawn collections from native populations in these lakes in Algonquin Provincial Park (southcentral Ontario; 45°47′N, 78°12′W). Both populations are known to be native (Wilson and Hebert, 1996, 1998; Halbisen and Wilson, 2009), are well represented in the literature (Morbey *et al.*, 2006, 2010; Dunlop *et al.*, 2010), and show contrasting growth and sensitivity to increased temperatures in controlled conditions (McDermid *et al.*, 2010, 2013). The Lake Manitou population originated from Lake Manitou on Manitoulin Island (45°45′N, 81°57″W), and has been used by the Ontario Ministry of Natural Resources (OMNR) as a source for fish stocking for over 50 years, using fresh wild spawn collections once per generation (OMNR, 2005). The Myrt Lake population originated from wild spawn collections from Myrt Lake in northwestern Ontario (48°26′N, 90°43″W) in 2009. Myrt Lake is a small (273 ha) meromictic lake with limiting oxygen and temperature conditions in the hypolimnion and epilimnion, respectively, limiting lake trout to the metalimnion for most of the year (J. McDermid, unpublished data). Although no genetic data have been collected for this population, lake trout in Myrt Lake are presumed to be native, because no stocking records exist for this lake (OMNR stocking database).

All experimental fish used from each population were one generation removed from the wild (their parents were collected as eggs from wild populations). Within each population, the number of fish per family was equalized at each life stage to ensure equal representation and minimize captive selection effects. Given that all families in each population were equally represented and only one generation removed from the wild, there had been no opportunity for transgenerational adaptation within any of the populations.

### Experimental subjects and husbandry

Adults from all strains were spawned in the autumn of 2009 using single-pair crosses (mating one male with one female) in the OMNR Codrington Fisheries Research Facility (Codrington, ON, Canada). This facility is supplied year-round with water through a flow-through water source (stream) and a holding pond created by a dam. Eggs were reared in family-specific lots in identical partitioned Heath trays until hatching. Hatched sac fry were transferred from Heath trays to replicate tanks and maintained in family-specific lots. Each population was represented by four families, which were kept separate but reared in common controlled conditions through all life stages prior to initiation of the thermal acclimation treatments. In November 2011, 2-year-old lake trout from all four populations received population-specific tags using visible implant elastomers (VIE; Northwest Marine Technologies, Shaw Island, WA, USA). No effort was made to identify individual families within strains; although tracking family-specific information would have been desirable, this was not possible due to logistical constraints.

### Experimental design

Immediately after tagging, equal numbers of fish (304 individuals per population) were randomly and evenly divided (38 fish per population per tank) among eight 200 l thermal acclimation tanks (Frigid Units Inc., Toledo, OH, USA) at ambient water temperature (∼8°C). In this way, fish from all four populations were held in identical conditions for each rearing temperature, but were distinguishable based on VIE markings. Tanks were then randomly assigned to one of four target temperatures (8, 11, 15 and 19°C; Table 1), with two acclimation tanks assigned to each treatment (76 individuals per population per acclimation temperature). These acclimation temperatures were chosen based on existing ecological and physiological literature on lake trout. For example, 8°C is a typical summer hypolimnetic temperature inhabited by juvenile lake trout (Plumb and Blanchfield, 2009), 11°C is within the optimal temperature range for growth (O'Connor *et al.*, 1981; Christie and Regier, 1988) and the species' preferred temperature range of 10–12°C (McCauley and Tait, 1970; McDermid *et al.*, 2013), aerobic metabolism is maximized at ∼15°C, and 19°C is warmer than the thermal optimum for lake trout, but below the incipient lethal temperature (Gibson and Fry, 1954; Evans, 2007).

**Table 1:** Desired and achieved acclimation temperatures for eight acclimation tanks prior to critical thermal maximum (CTM) and metabolic rate experiments, showing mean values ± SEM for each acclimation temperature and tank

Desired acclimation temperature (°C)	Acclimation tank	Mean temperature before CTM (°C)	Mean temperature before respirometry (°C)
8	1	8.75 ± 0.013	8.47 ± 0.009
	2	8.75 ± 0.013	8.43 ± 0.009
11	3	11.02 ± 0.008	11.01 ± 0.005
	4	10.87 ± 0.008	11.01 ± 0.003
15	5	15.01 ± 0.004	15.17 ± 0.005
	6	15.23 ± 0.005	15.17 ± 0.002
19	7	18.68 ± 0.007	19.04 ± 0.003
	8	19.29 ± 0.003	19.27 ± 0.003

In May and June of 2012, fish were gradually acclimated to the four target temperatures from ambient temperature at a rate of 1°C day^−1^ (Underwood *et al.*, 2012). Water temperatures were increased using programmable heaters (Finnex TH-0300 and TH-0500, Chicago, IL, USA). Acclimation temperatures were maintained ±1.0°C for a minimum of 4 weeks before trials began. Water quality was maintained using a partial recirculation system (80% recirculation) through water filtration pumps (Eheim; Dollard Des Ormeaux, QC, Canada), which were cleaned weekly. Water circulation, temperature homogeneity and adequate oxygenation (>6 mg l^−1^) were obtained in each tank by bubbling compressed air through 58 cm air stones. Water temperature was monitored with Tidbit V2 Water Temperature loggers (Onset HOBO data loggers, Pocasset, MA, USA). Fish were fed to satiation twice daily using 3.0 mm Optimum Salmonid feed (COREY Nutrition Co., Fredericton, NB, Canada). Experimental fish were food deprived for 24 h prior to thermal resistance and respirometry experiments.

### Critical thermal maximum

Following 4 weeks of thermal acclimation, interpopulation variation in upper thermal resistance was assessed at each acclimation temperature using the critical thermal maximum (CTM) methodology (Becker and Genoway, 1979) following the protocol of Stitt *et al.* (2014). Using VIE markings to identify population, groups of randomly selected fish from each population were introduced into two 172 l thermal challenge tanks (five fish per population per tank; 20 fish per tank) at their acclimation temperature (8, 11 15, or 19°C). Fish from the different populations were randomly selected from within the acclimation tanks at a given acclimation temperature, such that fish from the different populations at a shared acclimation temperature were tested together. Following overnight acclimation to the thermal challenge tanks, water temperature was increased by 0.17°C min^−1^ until fish exhibited loss of equilibrium, defined by the inability to maintain dorsoventral orientation. Each fish was individually removed from the thermal challenge tank upon any sign of loss of equilibrium. Temperature was controlled by balancing the inflow of ambient water (∼8°C for the 8°C acclimation group; 11°C for the 11, 15 and 19°C acclimation groups) and the flow of hot water (∼60°C) through four aluminum-plated heating elements at the base of the tank. Dissolved oxygen levels were monitored using a YSI Pro dissolved oxygen probe (Hoskin Scientific, Burlington, ON, Canada) (±0.2 mg l^−1^) and maintained above 6 mg l^−1^ using three 25 cm air stones in each tank. The aeration also ensured mixing so that the water temperature was evenly distributed throughout the tanks. Upon loss of equilibrium, each fish was immediately removed and anaesthetized in a 100 mg l^−1^ buffered MS-222 solution (Sigma-Aldrich, St Louis, MO, USA) in order to obtain length and weight measurements and population identification. The thermal ramping rate used in this study was slower than what is typically used (e.g. 0.33°C min^−1^; Fangue *et al.*, 2006) to accommodate the high sensitivity of lake trout to temperature change (McDermid *et al.*, 2013). A slower rate of 0.17°C min^−1^ was therefore preferred, because this allowed for the recovery of all fish following loss of equilibrium.

### Respirometry design

Following 4 months of thermal acclimation, routine and maximal metabolic rate were measured individually for 160 lake trout of ∼100 g (10 fish per population per acclimation temperature) using four custom-built 1.1 l (3.5 cm internal diameter, 29.5 cm in length) cylindrical glass respirometers connected to four separate flow-through respirometry systems with independent flow controls. Metabolic rates were measured at the four acclimation temperatures (8, 11, 15 and 19°C). All respirometry equipment was submerged in two 100 l water-baths at acclimation temperature ±0.3°C (two respirometers per tank). Water temperature in the baths was controlled by balancing the inflow of ambient water (∼7°C for the 8°C acclimation group; 8–10°C for the 11, 15 and 19°C acclimation groups) with two 500 W heaters (Finnex TH-0500) connected to separate temperature controllers (Finnex HC-0810M). Each respirometry system contained a glass chamber connected to two water pumps (Marineland MaxiJet-400, Blacksburg, VA, USA) via gas-proof tubing. One pump recirculated water from the chamber past a dissolved oxygen probe (Vernier Technologies S120, Beaverton, OR, USA) and back, while the second pump flushed the chamber at a rate of 4.5 l min^−1^ with fresh, oxygenated water and returned it to the water-bath through a tube elevated above the water surface. Oxygen consumption rate was determined by turning off the flush pump to each chamber and measuring the decline in oxygen over 5 min. Dissolved oxygen levels were maintained above 6 mg l^−1^ in a similar manner to CTM experiments. Dissolved oxygen probes were connected to a Vernier Technologies Lab Pro (Vernier Software and Technology, Beaverton, OR, USA). The data from the Lab Pro were downloaded into LoggerPro software (version 3.8.6; Vernier Software and Technology), and the rate of decline in water O_2_ content (in milligrams of oxygen per litre per minute) was determined by fitting a linear regression to the O_2_–time data. Oxygen consumption (MO_2_) was then calculated using the following formula:

$$\begin{array}{rl} & {\text{MO}}_{2}({\text{in milligrams of O}}_{2}\text{ per hour})\\ & \;=\text{Rate of decline in}[{\text{O}}_{2}]\times (V-V_{m})\times 60 \end{array}$$

where *V* is respirometer volume (in litres) and *V*_m_ is the volume of the fish (in litres). All probes were calibrated daily against an anoxic solution of sodium sulfite and fully aerated water from the experimental tank. This set-up allowed for the simultaneous measurement of metabolic rate in four juvenile lake trout at their respective acclimation temperatures (8, 11, 15 or 19 ± 0.3°C). All respirometry trials were conducted in the autumn of 2012.

### Respirometry protocol

As fish held at higher temperatures exhibited more rapid growth, trials were run on fish from tanks in decreasing order of acclimation temperatures (19°C first, followed by 15, 11 and 8°C). The order of acclimation temperatures should not affect the results because all fish were provided with a minimum of 3 months to acclimate thermally to their respective treatments. Within acclimation temperatures, the order of populations was randomized. The evening before each experiment, individual fish were randomly selected from the acclimation tanks and placed individually into each respirometer at their acclimation temperature (±0.3°C) for overnight recovery from handling (12–14 h). Preliminary experiments showed that this period of recovery was sufficient to minimize the effect of transfer and handling stress on lake trout metabolic rate (N. I. Kelly, unpublished data). Observations were made of the fish in the chambers once they were placed into the chamber (before the overnight recovery period) and after all measurements were made the next day. Fish were always sitting on the bottom of the respirometer with no sign of agitation and were not observed swimming against the water flow. Respirometry chambers were covered with black cloth to provide a darkened environment and reduce disturbance. Following the overnight acclimation period, oxygen consumption measurements were performed over 30 min for each fish, starting at 08 00 h each day. Oxygen consumption was estimated as the average of the slopes obtained from the three regression lines. This average was used as an index of routine metabolic rate (RMR), the mean rate of oxygen consumption recorded in a fish in experimental conditions allowing only random activity and protection from external stimuli (Fry, 1971).

Maximal metabolic rate (MMR) is the maximal oxygen consumption rate recorded in a fish during or after exhaustive exercise (Fry, 1971; Roche *et al.*, 2013). To estimate MMR in juvenile lake trout, we used a ‘chase protocol’ (Cutts *et al.*, 2002; Norin and Malte, 2011; Healey and Schulte, 2012). Chase protocols are biochemically and physiologically representative of exhaustive exercise (Reidy *et al.*, 1995), although they may also elicit significant contributions of anaerobic metabolism to MMR estimates (Scarabello *et al.*, 1992). The use of the chase protocol to estimate maximal oxygen consumption rate and calculate metabolic scope has been outlined by Clark *et al.* (2013) and has been used in many published physiological studies (e.g. Norin and Malte, 2011; Healey and Schulte, 2012; Roche *et al.*, 2013). This protocol can be particularly useful for estimating maximal oxygen consumption in species such as lake trout, which do not rely on sustained swimming and, instead, use burst swimming to ambush prey species while foraging (M. Ridgway, personal communication)*.* Approximately 1 h after RMR measurements, fish were individually transferred from their respective respirometry chamber into a 200 l tank containing oxygenated (>90% saturation) water at acclimation temperature. Using a combination of hand chasing and light tail pinching, fish were individually chased until exhaustion (∼4.5–5 min). Fish generally displayed burst swimming for the first 1–1.5 min, followed by 2–3 min of slower swimming with infrequent burst activity and finally 1–2 min of slow swimming until exhaustion, when the fish became unresponsive to physical stimulation. Upon exhaustion, each fish was transferred quickly (∼10–15 s) back into the respirometry chamber for eight 5 min oxygen consumption measurements over the following 75 min. Each measurement period was separated by a 5 min flush period, during which the fish received fresh, oxygenated water from the water-bath at 4.5 l min^−1^. The eight post-chase oxygen consumption measurements per fish were plotted over time and fitted with a logarithmic curve. The *y*-intercept of this curve was taken as an estimate of MMR, i.e. the oxygen consumption rate of the fish immediately after exhaustion. By extrapolating to the intercept, we accounted for the decline in metabolic rate that occurs between the end of the chase and the first measurement in the respirometer. The MMR data were also analysed by estimating MMR as the highest value obtained post-chase for each fish (without back calculating, *sensu* Roche *et al.*, 2013); however, the results of the statistical analysis were very similar. We therefore followed Brett (1964), and only the back-calculated MMR data are presented here. The quantitative difference between RMR and MMR was considered as the metabolic scope for activity (Clark *et al.*, 2013).

Once metabolism measurements were completed, each fish was killed in a 250 mg l^−1^ solution of buffered MS-222 (Sigma-Aldrich), weighed, measured and identified to population. To account for bacterial oxygen consumption, 5 min measurements were made daily without fish in each respirometry chamber after experimental trials were completed, and these values were subtracted from those obtained during RMR and MMR measurement periods.

### Statistical analyses

All data met assumptions of normality. Metabolic variables (RMR, MMR and metabolic scope for activity), body mass and condition factor were log_10_ transformed to meet the assumption of homogeneity of variance. The effects of population and acclimation temperature on thermal resistance (CTM) were examined using a two-factor analysis of variance (ANOVA). Metabolic rate variables (RMR, MMR and metabolic scope) were examined using a two-factor analysis of covariance (ANCOVA) with body mass as a covariate. The metabolic rate data were corrected to a 108.6 g lake trout, the overall mean body weight of the fish used in these experiments. The mass exponents for RMR, MMR and metabolic scope were 1.12, 0.82 and 0.71, respectively. All variables passed the test of homogeneity of regression slopes for body mass. To assess variation among populations and acclimation temperatures in the metabolic recovery from exhaustive exercise, the logarithmic regression slope values (decline in metabolic rate over time) used to back-calculate MMR were examined with a two-factor ANCOVA with body mass as a covariate. As we were interested in whether populations differed in their capacity for acclimatization, we tested for a significant interaction between population and acclimation temperature for all variables. For the MMR chase trials, the effects of population and acclimation temperature on the time until exhaustion were tested with a two-factor ANOVA. We tested for a significant effect of acclimation temperature and population on body mass and fork length for CTM and metabolic rate variables using a two-factor ANOVA. For the metabolic rate data, condition factor was calculated for each fish and analysed with a two-factor ANOVA. To account for variation between replicate acclimation tanks, we also performed a linear mixed model with replicate acclimation tank as a random effect. As this analysis did not change our conclusions, we present the results from the ANCOVA model.

All statistical tests were performed using R (version 2.15.1 for Mac, available at http://www.r-project.org) and *P* < 0.05 as the level of significance. In the event of a significant main effect (significant effect of acclimation temperature or population of origin), Tukey's honestly significant difference (HSD) test was used to determine where the differences occurred. All thermal resistance and metabolic rate values presented are adjusted least-squares means ± SEM using body mass as a covariate.

## Results

### Physical characteristics of lake trout populations

For the thermal resistance experiments, there was no difference among populations in either body mass or fork length (Table 2; *F*_3,144_ = 2.03, *P* = 0.11 and *F*_3,144_ = 2.68, *P* = 0.05, respectively). Body mass and size did vary across acclimation temperatures (Table 2; *F*_3,144_ = 4.01, *P* < 0.01 and *F*_3,144_ = 6.28, *P* < 0.001 for body mass and fork length, respectively). Lake trout acclimated to 15°C weighed significantly less than fish acclimated to 11°C (Tukey's HSD, *P* < 0.05). Lake trout acclimated to 15°C were significantly smaller than fish acclimated to 8 or 11°C, and fish acclimated to 19°C were smaller than fish acclimated to 8°C (Tukey's HSD, *P* < 0.05). There was no significant interaction between population and acclimation temperature for body mass or fork length (*F*_9,144_ = 0.63, *P* = 0.77 and *F*_9,144_ = 0.97, *P* = 0.47, respectively).

**Table 2:** Body mass (in grams), fork length (in centimetres) and condition of four lake trout populations at four acclimation temperatures at the start of upper thermal resistance and respirometry experiments, showing sample sizes measured (*N*) and means ± SEM

Acclimation temperature (°C)	Population	Thermal resistance				Respirometry			
		*N*	Mass (g)	Fork length (cm)	Condition factor (*K*)	*N*	Mass (g)	Fork length (cm)	Condition factor (*K*)
8	Myrt Lake	10	58.6 ± 5.2	16.9 ± 4.6	1.1 ± 0.04	9	116.9 ± 3.8	20.7 ± 2.3	1.3 ± 0.01
	Lake Louisa	10	52.0 ± 7.1	16.3 ± 7.1	1.1 ± 0.02	10	114.4 ± 4.4	21.1 ± 2.4	1.2 ± 0.05
	Opeongo Lake	10	54.0 ± 5.6	16.9 ± 5.7	1.1 ± 0.01	10	111.4 ± 4.7	21.1 ± 3.2	1.2 ± 0.01
	Lake Manitou	10	51.7 ± 3.7	17.1 ± 3.7	1.0 ± 0.02	10	112.3 ± 3.9	21.6 ± 2.8	1.1 ± 0.02
11	Myrt Lake	10	53.0 ± 4.1	16.2 ± 4.8	1.2 ± 0.02	9	111.7 ± 6.4	20.9 ± 3.3	1.2 ± 0.04
	Lake Louisa	10	52.9 ± 4.7	16.4 ± 4.8	1.2 ± 0.02	10	116.5 ± 5.4	21.4 ± 2.7	1.2 ± 0.05
	Opeongo Lake	10	59.9 ± 3.0	17.5 ± 3.1	1.1 ± 0.02	10	115.9 ± 5.5	21.3 ± 3.1	1.2 ± 0.02
	Lake Manitou	10	53.3 ± 5.0	16.9 ± 4.7	1.1 ± 0.03	10	120.7 ± 4.7	22.0 ± 3.1	1.1 ± 0.02
15	Myrt Lake	10	52.2 ± 5.9	16.1 ± 5.2	1.2 ± 0.03	10	116.5 ± 4.0	20.4 ± 2.4	1.4 ± 0.03
	Lake Louisa	10	33.7 ± 5.6	14.1 ± 7.8	1.1 ± 0.02	10	120.8 ± 7.4	21.6 ± 3.4	1.2 ± 0.03
	Opeongo Lake	10	48.0 ± 4.5	15.9 ± 5.1	1.2 ± 0.02	10	111.7 ± 4.6	20.8 ± 4.6	1.3 ± 0.04
	Lake Manitou	10	46.2 ± 5.2	16.2 ± 5.4	1.0 ± 0.03	10	115.2 ± 5.2	21.3 ± 2.9	1.2 ± 0.02
19	Myrt Lake	10	47.0 ± 5.0	15.6 ± 4.2	1.2 ± 0.05	10	88.8 ± 8.0	18.3 ± 4.0	1.4 ± 0.04
	Lake Louisa	10	44.1 ± 4.9	15.9 ± 5.6	1.1 ± 0.04	10	84.3 ± 5.9	18.6 ± 4.5	1.3 ± 0.03
	Opeongo Lake	10	51.5 ± 4.5	16.0 ± 4.7	1.2 ± 0.03	10	98.5 ± 5.5	19.2 ± 4.2	1.4 ± 0.06
	Lake Manitou	10	43.2 ± 2.5	15.9 ± 2.1	1.1 ± 0.03	10	84.1 ± 6.7	19.1 ± 4.3	1.2 ± 0.02

For fish used in the respirometry experiments, populations did not vary significantly in body mass (*F*_3,142_ = 0.12, *P* = 0.95), although significant differences in fork length were observed among the populations (*F*_3,142_ = 5.08, *P* < 0.01). Tukey's HSD comparisons showed that the Lake Manitou population was significantly longer than the Myrt Lake population (*P* < 0.01). Body mass and fork length of lake trout used in the respirometry experiments varied significantly among acclimation temperatures (*F*_3,142_ = 26.94, *P* < 0.001 and *F*_3,142_ = 51.61, *P* < 0.001, respectively), in that the lake trout acclimated to 19°C were significantly lighter and smaller than fish at all other acclimation temperatures (*P* < 0.001 for all). There was no significant interaction between population and acclimation temperature for body mass or fork length (*F*_9,142_ = 0.98, *P* = 0.46 and *F*_9,142_ = 0.64, *P* = 0.76, respectively). Condition factor varied significantly among populations (*F*_3,142_ = 20.80, *P* < 0.001). The Lake Louisa and Opeongo Lake populations were not significantly different from each other (*P* = 0.53); however, they were significantly fatter than the Lake Manitou population and leaner than the Myrt Lake population, respectively (*P* < 0.01 for all comparisons). Acclimation temperature had a significant effect on condition factor (*F*_3,142_ = 16.04, *P* < 0.001). Lake trout acclimated to 19°C were significantly fatter compared with all other acclimation temperatures (Tukey's HSD, *P* < 0.01 for all comparisons). There was no significant interaction between acclimation temperature and population for condition factor (*F*_9,142_ = 1.62, *P* = 0.12).

### Critical thermal maximum

**Figure 1:**
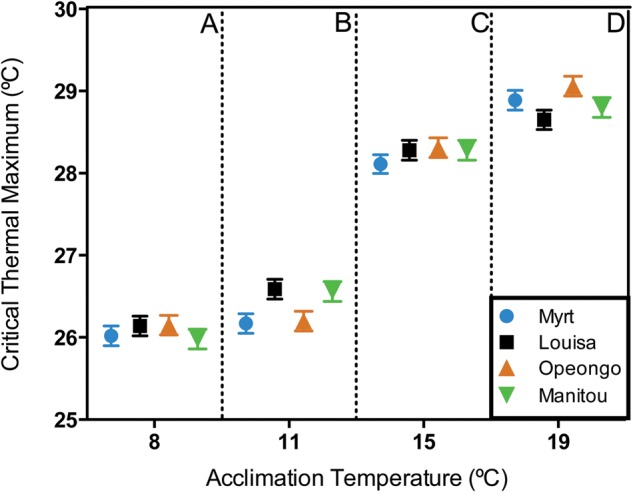
Thermal resistance (as measured by determining critical thermal maximum) of four lake trout populations acclimated to four temperatures (8, 11, 15 and 19°C). Values are least-squares means ± SEM. For acclimation temperatures, upper case letters indicate significant differences based on Tukey's honestly significant difference (HSD; *P* < 0.05).

The CTM of juvenile lake trout from all four populations increased significantly with acclimation temperature (Fig. 1; *F*_3,144_ = 487.18, *P* < 0.001). The CTM values differed significantly between all acclimation temperatures (Tukey's HSD, *P* < 0.01 for all comparisons). Across populations, lake trout acclimated to 19°C lost equilibrium at a temperature ∼3°C higher than did lake trout acclimated to 8°C (26.0–26.2 and 28.7–29.1°C for 8 and 19°C acclimation treatments, respectively; Fig. 1). The greatest increase in thermal resistance occurred between 11 and 15°C, where CTM for all populations increased by ∼1.5°C. Thermal resistance did not differ among the four lake trout populations studied (*F*_3,144_ = 0.95, *P* = 0.42; Fig. 1). There was no significant interaction between population and acclimation temperature for CTM (*F*_9,144_ = 1.76, *P* = 0.08), indicating that the four populations had similar capacity for thermal acclimation, at least over the range of temperatures studied.

### Routine metabolic rate

**Figure 2:**
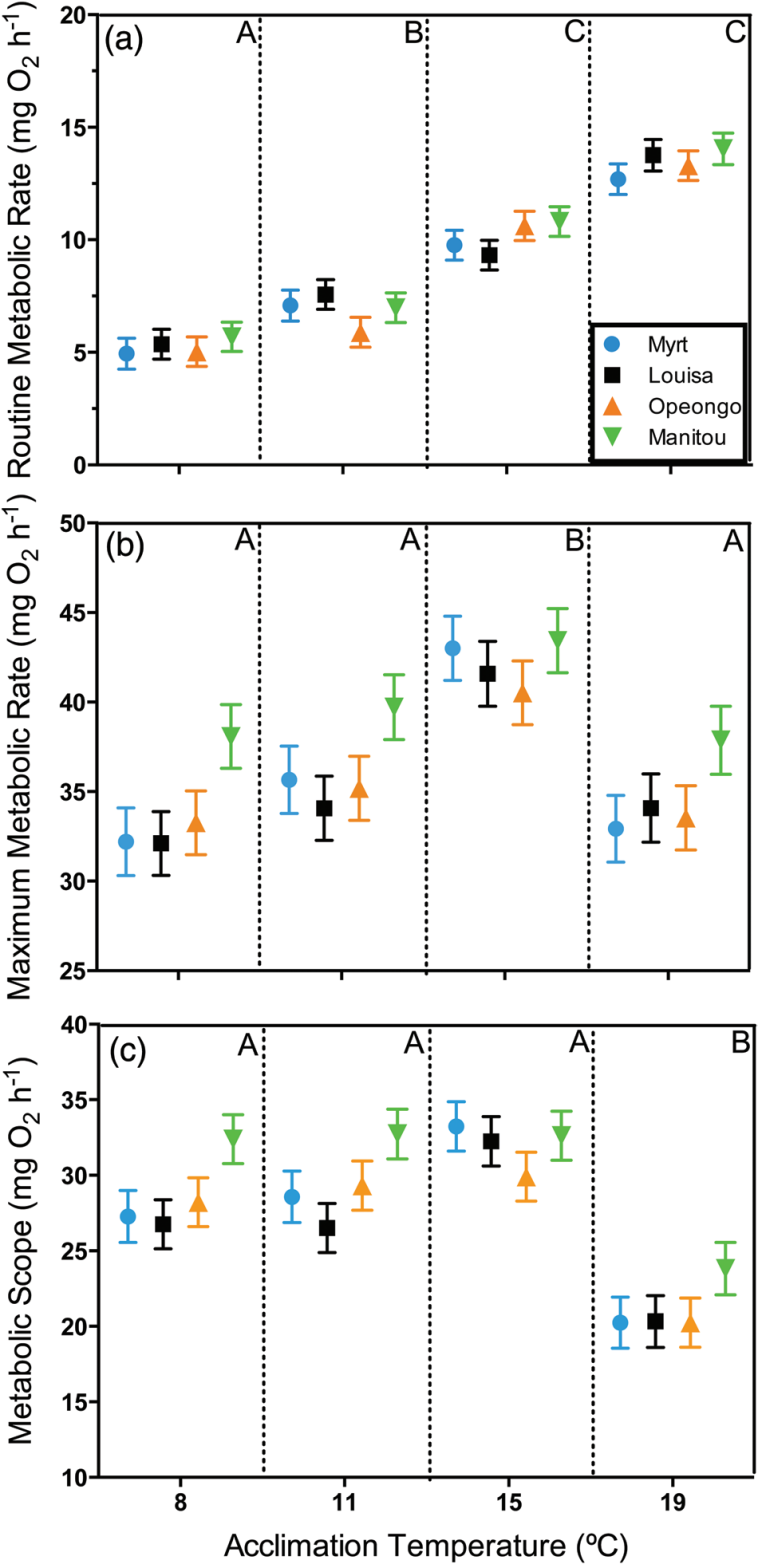
Variation in routine metabolic rate (RMR; **a**); maximal metabolic rate (MMR; **b**) and metabolic scope (**c**) represented by oxygen consumption (in milligrams of oxygen per hour) for four lake trout populations following 4 months of thermal acclimation to different temperatures. Metabolic scope was calculated by subtracting RMR from MMR. Values are least-squares means ± SEM adjusted for body mass (in grams) as a covariate. For acclimation temperatures, upper case letters indicate significant differences based on Tukey's HSD (*P* < 0.05).

Routine metabolic rate differed significantly with acclimation temperature (Fig. 2a; *F*_3,141_ = 123.20, *P* < 0.001; body mass covariate, *F*_1,141_ = 113.08, *P* < 0.001). Routine metabolic rate differed between all temperatures (Tukey's HSD, *P* < 0.001 for all comparisons), with the exception of 15 and 19°C, which did not differ significantly from each other (*P* = 0.08). The RMR of fish acclimated to 19°C (12.69–14.05 mg O_2_ h^−1^) was ∼3-fold higher than for fish acclimated to 8°C (4.94–5.69 mg O_2_ h^−1^; Fig. 2a). Similar to patterns of CTM, RMR did not differ significantly among the four lake trout populations (Fig. 2a; *F*_3,141_ = 1.36, *P* = 0.26). There was no significant interaction between population and acclimation temperature for RMR (*F*_9,141_ = 0.87, *P* = 0.55).

### Maximal metabolic rate

Maximal metabolic rate varied significantly with acclimation temperature (Fig. 2b; *F*_3,141_ = 15.66, *P* < 0.001; body mass covariate, *F*_1,141_ = 128.08, *P* < 0.001). The MMR of all populations increased by 20–30% between 8 and 15°C (32.11–38.07 and 40.53–43.43 mg O_2_ h^−1^ at 8 and 15°C, respectively), the temperature at which MMR was highest (*P* < 0.001 for all comparisons). Above 15°C, the MMR for all populations declined (Tukey's HSD, *P* < 0.001; Fig. 2b). Maximal metabolic rate differed significantly among lake trout populations (*F*_3,141_ = 6.78, *P* < 0.001; Fig. 2b). The Lake Manitou population had a significantly higher MMR than the three other populations (Tukey's HSD, *P* < 0.01 for all comparisons). There was no significant interaction between population and acclimation temperature for MMR (*F*_9,141_ = 0.55, *P* = 0.83).

### Metabolic scope for activity

The metabolic scope (MMR minus RMR) differed significantly with acclimation temperature (Fig. 2c; *F*_3,141_ = 28.00, *P* < 0.001; body mass covariate, *F*_1,141_ = 55.37, *P* < 0.001). Metabolic scope at 8, 11 and 15°C was not significantly different (Tukey's HSD, *P* > 0.05 for all comparisons). At 19°C, all populations displayed a significant decline in metabolic scope for activity when compared with all other acclimation temperatures (Tukey's HSD, *P* < 0.001 for all comparisons). There was a significant difference in metabolic scope among lake trout populations (Fig. 2c; *F*_3,141_ = 5.37, *P* < 0.01). Comparable to patterns for MMR and metabolic recovery (see subsection below, ‘*Metabolic recovery from exhaustive exercise*’), the Lake Manitou population had a significantly higher metabolic scope than the other three populations (Tukey's HSD, *P* < 0.05 for all comparisons). There was no significant interaction between population and acclimation temperature for metabolic scope (*F*_9,141_ = 0.67, *P* = 0.74).

### Time until exhaustion for chase protocol

**Figure 3:**
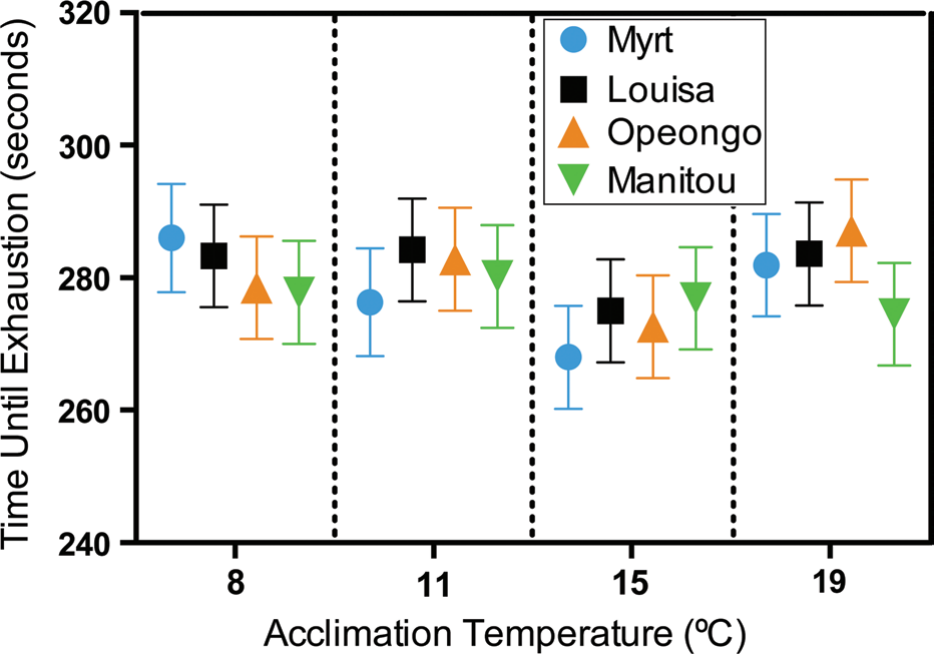
Time until exhaustion for four lake trout populations acclimated for 4 months to four temperatures from chase trials to measure MMR. Values are least-squares means ± SEM.

There was no significant effect of population or acclimation temperature on the time until exhaustion during chase protocols to estimate MMR (Fig. 3; *F*_3,142_ = 0.25, *P* = 0.86 and *F*_3,142_ = 01.13, *P* = 0.34 for population and acclimation temperature, respectively). There was no significant interaction between population and acclimation temperature (*F*_9,144_ = 0.30, *P* = 0.97).

### Metabolic recovery from exhaustive exercise

**Figure 4:**
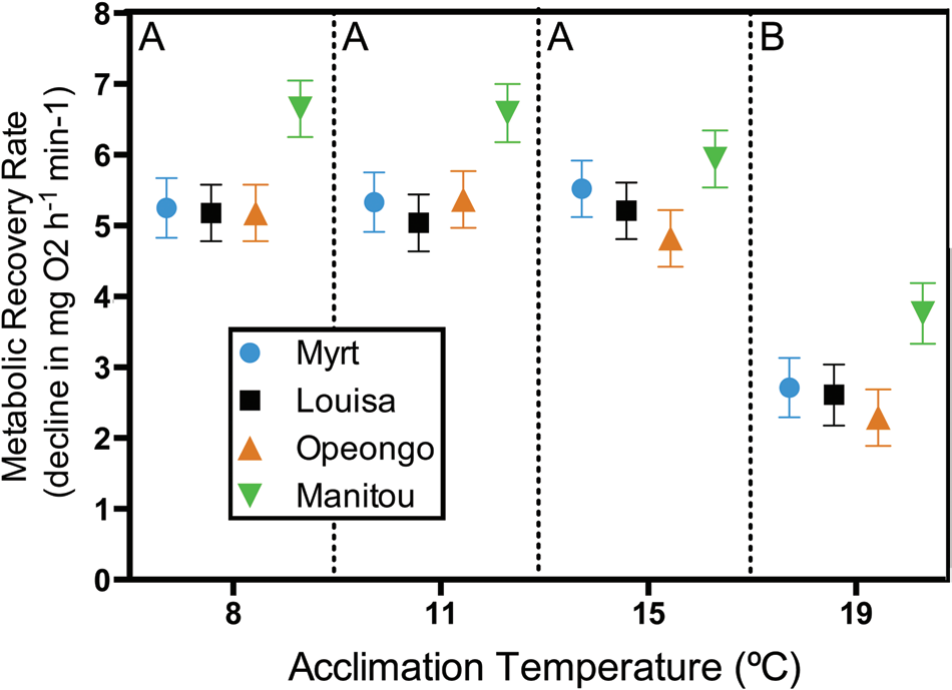
Variation in the metabolic recovery rate represented by the decline in oxygen consumption (in milligrams of oxygen per hour) over time (per minute) for four lake trout populations following exhaustion via a chase protocol. Values are least-squares means ± SEM adjusted for body mass (in grams) as a covariate. For acclimation temperatures, upper case letters indicate significant differences based on Tukey's HSD (*P* < 0.05).

The metabolic recovery after exhaustive exercise, estimated by the decline in metabolic rate over time following a chase protocol, differed significantly with acclimation temperature (Fig. 4; *F*_1,141_ = 28.24, *P* < 0.001; body mass covariate, *F*_1,141_ = 12.56, *P* < 0.001). Lake trout acclimated to 19°C had significantly lower metabolic recovery rates than those at all other acclimation temperatures (Tukey's HSD, *P* < 0.001 for all comparisons). There was also a significant effect of population on recovery rate (Fig. 4; *F*_1,141_ = 8.85, *P* < 0.001). The metabolic recovery rate was significantly higher for the Lake Manitou population compared with all other populations (Tukey's HSD, *P* < 0.01 for all comparisons). There was no significant interaction between population and acclimation temperature for the metabolic recovery rate (*F*_9,141_ = 0.37, *P* = 0.95).

## Discussion

### Interpopulation variation

There was no evidence of interpopulation variation in upper thermal resistance among the four lake trout populations examined in this study, despite their reciprocal isolation since postglacial colonization. In contrast, McDermid *et al.* (2013) reported significant variation in thermal preference and CTM between juvenile lake trout from Lake Louisa and Opeongo Lake reared in a common environment. However, CTM differences in that study were small (<0.5°C) and probably not biologically significant. The lack of variation for CTM among lake trout populations in the present study suggests that the upper thermal limits of lake trout are highly conserved across the populations represented in our study.

The lack of interpopulation variation in CTM reported in the present study adds to the conflicting results from previous studies of physiological variation in thermal performance among salmonid populations. Interpopulation variation in CTM has been reported for other salmonid species, including sockeye salmon (*O. nerka*; Eliason *et al.*, 2011), cutthroat trout (*O. clarkii pleuriticus*; Underwood *et al.*, 2012) and brook trout (*S. fontinalis*; McDermid *et al.*, 2012; Stitt *et al.*, 2014). In contrast, our results are congruent with other studies that suggest that the thermal performance of salmonids can remain highly conserved among populations (Jensen *et al.*, 2000; Myrick and Cech, 2000; Rodnick *et al.*, 2004; Larsson *et al.*, 2005; Elliott and Elliott, 2010).

There was limited evidence of metabolic variation among lake trout populations in this study. The RMR of juvenile lake trout did not vary among populations and, when converted to mass-specific metabolic rate (in milligrams of oxygen per kilogram per hour), are comparable to those summarized by Evans (2007) for juvenile lake trout from various populations (Gibson and Fry, 1954; Stewart *et al.*, 1983; Beamish *et al.*, 1989; Rottiers, 1993). The mean mass-specific RMR of 108.6 g lake trout acclimated to 8°C in our study of 48.41 mg O_2_ kg^−1^ h^−1^ is within the range of standard metabolic rate values reported for 100 g lake trout by Gibson and Fry (1954) of 36.56 mg O_2_ kg^−1^ h^−1^ and by Stewart *et al.* (1983) of 74.87 mg O_2_ kg^−1^ h^−1^. In contrast to RMR, the Lake Manitou population had a higher metabolic capacity, as indicated by their significantly higher MMR and metabolic scope for activity compared with the other populations. This could have management implications, because the Lake Manitou population has been used for supplemental stocking into inland lake trout populations in Ontario (OMNR, 2005; Halbisen and Wilson, 2009). However, the MMR and metabolic scope values for the other populations were very similar. The mass-specific MMR values reported here for 108.6 g lake trout differ from active metabolic rate (AMR) values reported by Gibson and Fry (1954) for 100 g fish by approximately +50.6, +24.5, +3.4 and −9.9% at 8, 11, 15 and 19°C, respectively. The large discrepancy between studies at 8 and 11°C may be due to the chase protocol, which relies on short-duration burst swimming activity, primarily supported by anaerobic metabolism. Thus, MMR values reported here correspond to the combination of each fish's RMR and excess post-exercise oxygen consumption to repay the oxygen debt accumulated from anaerobic metabolism during the chase (Killen *et al.*, 2007). Maximal oxygen consumption rates estimated from chase protocols can be much higher than those produced by sustained swimming speed protocols typically used to estimate AMR (Reidy *et al.*, 1995; but see Roche *et al.*, 2013). The similarity between our MMR values and those of AMR (Gibson and Fry, 1954), respectively, at 15 and 19°C, could be due to either a decline in anaerobic metabolism at higher acclimation temperatures or population-specific variation in MMR.

Compared with the numerous studies that have investigated interpopulation variation in thermal stress, there is relatively little information about such variation in metabolic rate. Interpopulation variation in AMR and metabolic scope has been reported for coho salmon (*Oncorhynchus kisutch*; Lee *et al.*, 2003) and sockeye salmon (Lee *et al.*, 2003; Eliason *et al.*, 2011), and population-specific variation in standard metabolic rate has been reported for brown trout (*Salmo trutta*; Lahti *et al.*, 2002; Cano and Nicieza, 2006), brook trout (Stitt *et al.*, 2014) and largemouth bass (*Micropterus salmoides*; Cooke *et al.*, 2001). The limited variation in metabolism among lake trout populations provides insights into the adaptive capacity of physiological systems at the population level.

The lake trout populations in this study exhibited a similar capacity for phenotypic plasticity of CTM and metabolism upon thermal acclimation. Likewise, McDermid *et al.* (2013) reported no significant interaction between acclimation temperature and lake trout population for CTM. This contrasts with previous studies, which have largely found that the thermal acclimatization capacity varies among populations within a species, including metabolic enzyme activity (cytochrome *c* oxidase, Lucassen *et al.*, 2006; citrate synthase and lactate dehydrogenase, Seebacher *et al.*, 2012), swimming performance (Sylvestre *et al.*, 2007; Seebacher *et al.*, 2012; Stitt *et al.*, 2014), RMR and thermal resistance (Stitt *et al.*, 2014). Variation among populations in their capacity for thermal acclimatization could be advantageous for species facing environmental change across wide geographical ranges, because some populations would be better suited to cope with novel environmental conditions (Somero, 2010). The lack of interpopulation variation in thermal acclimatization capacity, combined with the limited variation observed among populations for thermal resistance and metabolism, suggests that predicted climate-induced temperature elevations could have significant impacts on lake trout populations across a wide geographical range.

### Effect of thermal acclimation

Thermal acclimation had a significant effect on lake trout thermal resistance. This agrees with previous studies where the CTM of juvenile lake trout increased from ∼25.5 to 28.5°C (McDermid *et al.*, 2013; Opeongo Lake and Lake Louisa populations), and the time to 50% mortality increased across a similar acclimation temperature range (8–20°C; Gibson and Fry, 1954). The CTM values reported for juvenile Opeongo Lake and Lake Louisa lake trout by McDermid *et al.* (2013) agree with those reported here (Fig. 1). Phenotypic plasticity is clearly a mechanism by which the thermal limits of lake trout can adjust to changes in environmental temperature.

The effect of acclimation temperature on lake trout upper thermal resistance was comparable to CTM–acclimation temperature relationships demonstrated for other salmonids (Lee and Rinne, 1980; Elliott and Elliott, 1995; Currie *et al.*, 1998; Myrick and Cech, 2005; Underwood *et al.*, 2012). A 2–3°C increase in CTM over an ∼10°C acclimation temperature range has been reported for steelhead trout (anadromous *Oncorhynchus mykiss*; Myrick and Cech, 2005), golden trout (*Oncorhynchus mykiss whitei*; Myrick and Cech, 2003) and brook trout (*S. fontinalis*; Lee and Rinne, 1980; Stitt *et al.*, 2014), but is relatively small compared with more eurythermal species (e.g. *Fundulus heteroclitus*, Fangue *et al.*, 2006). The reduced plasticity of CTM reported for salmonids in general suggests that these species may be particularly vulnerable upon exposure to chronically elevated temperatures.

Thermal acclimation also had a significant effect on the metabolism of the four lake trout populations. The RMR increased ∼3-fold over the acclimation temperature range. Above 15°C, all lake trout in the present study exhibited a decline in MMR. This decline in MMR, accompanied by the increase in RMR above 15°C, resulted in a sharp decline in metabolic scope at 19°C. The significant decline in both MMR and metabolic recovery rate above 15°C suggests that the maximal metabolic capacity of juvenile lake trout is impaired above this acclimation temperature. Likewise, previous studies have shown that the aerobic metabolism of juvenile lake trout peaks at 15–16°C and declines at higher acclimation temperatures (Gibson and Fry, 1954; Evans, 2007). Chronic temperature elevations could negatively affect juvenile lake trout metabolism through rising routine metabolic costs and reduced maximal metabolic capacity, potentially resulting in impaired juvenile growth and recruitment (McDonald *et al.*, 1996; Evans, 2007).

### Implications of climate change for coldwater species

Global climate change is predicted to have a significant impact on freshwater ecosystems and their resident biota (De Stasio *et al.*, 1996; Magnuson *et al.*, 1997; Schindler, 1997; Heino *et al.*, 2009). Empirical lake surface water temperature models based on current climate change scenarios project an increase of up to 18°C in water temperatures by 2100, with some lakes experiencing projected surface temperatures as high as 30°C (Sharma *et al.*, 2007). For small temperate lakes, climate models predict that elevated temperatures will alter water temperature profiles, increase the extent and duration of thermal stratification and reduce the amount of optimal thermal habitat for coldwater species (Stefan *et al.*, 1998; Ficke *et al.*, 2007). Empirical support for these predicted effects of increased atmospheric temperatures on lake water temperature profiles has been reported to some extent for temperate lakes (Schindler *et al.*, 1990; Snucins and Gunn, 2000). During summer thermal stratification, lake trout occupy the colder hypolimnion to seek refuge from warmer epilimnetic temperatures (Plumb and Blanchfield, 2009) and make short excursions into the warmer littoral zone to forage (Morbey *et al.*, 2006). Evidence from Yukon lakes also indicates that lake trout make use of cold upwellings as thermal refugia (Mackenzie-Grieve and Post, 2006). As mean epilimnetic water temperatures increase over the next century, populations of lake trout and other cold-adapted species will lose optimal thermal habitat and experience suboptimal temperatures for longer periods (Schindler *et al.*, 1990; Ficke *et al.*, 2007). This is predicted to have significant energetic consequences for lake trout populations, including potential impairments to metabolism (Evans, 2007), growth (Christie and Regier, 1988) and recruitment (McDonald *et al.*, 1996; Casselman, 2002). As a result, coldwater populations may become extirpated from major portions of their current geographical range (Casselman, 2002; Rahel, 2002; Chu *et al.*, 2005). For example, a 3°C increase in water temperature is predicted to result in a 20% reduction in the range and abundance of coldwater salmonids (Casselman, 2002).

The capacity for thermal acclimatization and adaptation will underlie the susceptibility of populations and species to climate change (Stillman, 2003; Calosi *et al.*, 2008; Somero, 2010). Thermal acclimatization of physiological traits will allow organisms to maintain performance and enhance fitness in novel environmental conditions (Wilson and Franklin, 2002), but this capacity is finite. Our data suggest that intraspecific variation for physiological traits within and among lake trout populations may be very limited, and that temperature increases may have regional as well as local impacts on lake trout populations.

Understanding how the multifaceted effects of climate change will impact lake trout populations is important for implementing successful management and conservation strategies. Apart from elevated temperatures, climate change is predicted to have a variety of negative effects on lake trout populations, including shifts in abundance of prey species and competition with warmwater species, such as smallmouth bass (Sharma *et al.*, 2007). Combined knowledge of intraspecific genetic structure and diversity (Wilson and Hebert, 1996, 1998), ecology (Morbey *et al.*, 2006; Dunlop *et al.*, 2010) and thermal physiology (Gibson and Fry, 1954; Evans, 2007; McDermid *et al.*, 2013; present study) can allow for better predictions concerning the response of this species to the predicted effects of climate change, particularly at the population level. In particular, vulnerability of different life stages to availability of suitable thermal habitat in both space and time (Evans, 2007; Shuter *et al.*, 2012) may require changes in fisheries management regulations or stocking programmes.

## Funding

This work was supported by funding from the Ontario Ministry of Natural Resources (OMNR), the Canada Foundation for Innovation, the Ontario Innovation Trust and The W. Garfield Weston Foundation.
